# Toward A Brain-Based Theory of Beauty

**DOI:** 10.1371/journal.pone.0021852

**Published:** 2011-07-06

**Authors:** Tomohiro Ishizu, Semir Zeki

**Affiliations:** Wellcome Laboratory of Neurobiology and Wellcome Department of Imaging Neuroscience, University College London, London, United Kingdom; Lund University, Sweden

## Abstract

We wanted to learn whether activity in the same area(s) of the brain correlate with the experience of beauty derived from different sources. 21 subjects took part in a brain-scanning experiment using functional magnetic resonance imaging. Prior to the experiment, they viewed pictures of paintings and listened to musical excerpts, both of which they rated on a scale of 1–9, with 9 being the most beautiful. This allowed us to select three sets of stimuli–beautiful, indifferent and ugly–which subjects viewed and heard in the scanner, and rated at the end of each presentation. The results of a conjunction analysis of brain activity showed that, of the several areas that were active with each type of stimulus, only one cortical area, located in the medial orbito-frontal cortex (mOFC), was active during the experience of musical and visual beauty, with the activity produced by the experience of beauty derived from either source overlapping almost completely within it. The strength of activation in this part of the mOFC was proportional to the strength of the declared intensity of the experience of beauty. We conclude that, as far as activity in the brain is concerned, there is a faculty of beauty that is not dependent on the modality through which it is conveyed but which can be activated by at least two sources–musical and visual–and probably by other sources as well. This has led us to formulate a brain-based theory of beauty.

## Introduction

In the work reported here, we address a question that has been addressed many times over past centuries, namely what constitutes beauty. The question was especially well formulated, in a neurobiologically accessible way, by Edmund Burke. In his *Philosophical Enquiry into the Origin of Our Ideas of the Sublime and Beautiful*, Burke wrote that “Beauty is, for the greater part, some quality in bodies acting mechanically upon the human mind by the intervention of the senses” [1]. That definition suggests that there is a unique faculty of beauty that can be stimulated by any and all the senses. It thus raises an important question: would the experience of beauty derived from different senses, say the visual and auditory, correlate with activity in the same or different brain areas? If the latter, then the clear implication would be that brain systems that correlate with the experience of beauty are functionally specialized, the experience of visual beauty correlating with activity in one area or set of areas and that of auditory beauty correlating with another. But our reading of the relevant humanistic literature, too numerous to mention, suggests that the first alternative has been more favored by those who have discoursed on the subject, namely that there is a single faculty of beauty into which different senses feed. This alternative is reflected in Burke's definition.

We thus sought to learn something, however small, about how brain activity might be organized during the experience of beauty. Burke and many others, including Immanuel Kant, Anthony Ashley-Cooper (3^rd^ Earl of Shaftesbury) and Joseph Addison, distinguished between the beautiful and the sublime, the latter having for them characteristics such as “awe”, “horror”, “disgust” and “fear”. In this work we are concerned with the beautiful alone, not the sublime. We undertook a human brain imaging experiment, using functional magnetic resonance imaging (fMRI), in which we asked subjects to view pictures of paintings and listen to brief musical excerpts and rate them according to how beautiful they seemed, while we imaged the activity in their brains. As a working hypothesis, we inclined more towards our neurobiological understanding of Burke's definition and supposed that there would be a single area or set of areas whose activity would correlate with the experience of beauty, regardless of whether it was derived from an auditory or visual source.

Previous work from this and other laboratories [2]–[6] has implicated activity in the mOFC-an acknowledged pleasure and reward center in the brain [7]-during the experience of visual or musical beauty but no equivalent study for the experience of beauty derived from two different senses in the same subjects has been reported. This is important, since the mOFC is a large expanse of cortex and different but sometimes overlapping parts of it have been activated by different tasks [5], [8]. We hypothesized that activity in the same part of mOFC would correlate with beauty in the more abstract sense, that is to say, regardless of whether it is derived from the auditory or visual sense. This turned out to be so and led us to formulate a brain-based theory of beauty.

## Materials and Methods

### Subjects

21 healthy right-handed volunteers (9 male, 12 female, mean age 27.5 years) participated in this study. All had normal or corrected-to-normal vision, and none had a history of neurological or psychiatric disorder. Written informed consent was obtained from all, and the study was approved by the Ethics Committee of the Institute of Neurology. All data was anonymized. Subjects were drawn from the following cultural groups: 10 West Europeans, 2 Americans, 4 Japanese, 3 Chinese and 2 Indian. Except for one subject, none was an artist or a musician.

### Psychophysical testing

Prior to scanning, psychophysical tests were used to select stimuli; this allowed subjects to classify stimuli into three groups-‘beautiful’, ‘indifferent’ and ‘ugly’-which were subsequently shown in the scanning sessions. We first tested 30 subjects (15 male, 15 female, mean age 25.8 years) who did not participate in the scanning. Each viewed 60 paintings and listened to 60 musical excerpts. The visual stimuli included paintings of portraits, landscapes and still lifes, most of them from Western art but three from Oriental art. The auditory stimuli included classical and modern excerpts of mainly Western music with two Japanese excerpts. All stimuli were presented for 16 s with an inter-trial interval of 2 s. Each stimulus was given a score from 1 to 9. Those given scores of 1–3 were classified as ‘ugly’, 4–6 were classified as ‘indifferent’ and 7–9 as ‘beautiful’. Based on the psychophysical testing, we selected 10 ‘beautiful’, 10 ‘indifferent’ and 10 ‘ugly’ stimuli in the visual and musical categories to be used for the scanning sessions, resulting in total of 60 stimuli (30 each for painting and music).

During a first visit to the laboratory, between one and two weeks prior to scanning, each subject was instructed about the experiment and rated the stimuli as described above. Only subjects classifying the stimuli into the three categories in roughly equal proportions were selected for the scanning experiment (see Supporting Information: Table S1. *Behavioral data collected in preliminary behavioral test*). One subject, who classified all visual stimuli as ugly or neutral, was excluded.

### Stimuli

Stimuli were generated using Cogent 2000 (http://www.vislab.ucl.ac.uk/cogent_2000) running in MATLAB (MathWorks, Natick, MA, USA). As a counterpart to the evolving and therefore dynamic nature of the musical stimuli, each visual stimulus was made to zoom continuously at the rate of 3° sec^−1^, using image-editing programs (Adobe^®^ Photoshop CS3^®^, Premiere Pro CS3^®^). The visual stimuli were back-projected onto a screen using a LCD projector through an angled mirror. The resolution of the screen was 1,400×1,050 pixels. Participants listened to the auditory stimuli through headphones (MR Confon, Magdeburg, Germany).

The session began with subjects viewing a flat black screen for 20 s to allow for T1 equilibration effects to subside (and the corresponding first six brain volumes were discarded). A fixation point was then presented at the center of the screen against a black background for 1 s. This was followed by the presentation of visual or auditory stimuli, in random order, for 16 s each, followed by an interval of 1 s. When musical stimuli were presented, a fixation point appeared at the center of the black screen and participants were asked to fixate it. After each stimulus presentation, participants were asked to rate them into one of three categories–of “beautiful”, “indifferent”, or “ugly”-using button presses with their right hand. As with the pre-scanning classification, we expressly asked subjects to rate their experience of the entire 16 s period during which they were exposed to the stimulus. The response period lasted for 5 s and participants could make their rating at any time during that period. The session ended with a blank period of 5 s, during which the scanner continued to acquire blood oxygen level-dependent (BOLD) signals. The stimuli were presented in 5 sessions, each consisting of 12 stimuli, half of which were auditory and the other half visual–presented in pseudo-random order. Each session contained three visual and three auditory stimuli. Prior to the scanning, participants had a short practice session using different visual and auditory stimuli to those used in the scanning session.

### Scanning details

Scanning data were acquired with a 3-T Siemens Magnetom Trio MRI scanner (Siemens, Erlangen, Germany) fitted with a 12-channel head-coil. An echo-planar imaging (EPI) sequence was applied for functional scans to obtain BOLD signal (echo time TE = 30 ms, repeat time TR = 70 ms, volume time 3.36 s) using 48 slices to cover the whole brain. The voxel resolution was 3 mm×3 mm in-plane resolution, with a 2 mm slice thickness and 1 mm inter-slice gap. T1-weighted anatomical images were acquired at the end of experimental sessions for each subject (176 slices, resolution 1×1×1 mm, TE = 2.48 ms, TR = 7.92 ms). We also recorded physiological responses, heart rate and breathing, for each subject.

### Analysis

All data were analysed using SPM8 (Statistical Parametric Mapping http://www.fil.ion.ucl.ac.uk/spm/software/spm8/). The EPI images for each subject were realigned and normalized into Montreal Neurological Institute (MNI) space, smoothed using Gaussian smoothing kernel of 9×9×9 mm, and filtered with a high-pass cutoff (128 s) to remove drift terms.

The stimulus for each subject was modelled as a set of regressors in a general linear model (GLM) first-level (within subject) analysis. The stimulus was a block design and boxcar functions were used to define stimulus functions, which modelled the onsets and durations of the appearances of each of the visual and musical stimuli. Key presses, modelled as delta functions, constituted an additional variable. Head movement parameters calculated from the realignment pre-processing step and physiological recordings were included as regressors of no interest. Stimulus functions were convolved with a canonical Hemodynamic Response Function (HRF) to provide regressors for the GLM. We carried out two separate analyses, categorical and parametric, encoding the same data in two different ways. For the categorical analysis, separate stimulus functions for beautiful, indifferent and ugly stimuli (based on the subject-specific responses) in each modality (visual and musical) were used. Contrast images for both musical and visual presentations and beauty ratings were taken to second-level (between subject) *t*-tests to produce statistical maps at the group level. We also analyzed our data for parametric modulation, for which visual and musical stimuli given a beauty rating were used as regressors, with beauty rating as the parametric modulator. Ratings were coded as -1, 0 and 1 for ‘ugly’, ‘indifferent’ and ‘beautiful’, and a 1^st^ order polynomial expansion was included.

Conjunction analyses [9] were used to characterize brain activations common to visual and musical experiences designated as beautiful or otherwise.

We report cluster level activations that were significant at *p*<0.05 corrected, although some of these were also significant at the voxel level at *p*<0.05 FWE (family wise error) corrected. In cases where we had a priori knowledge of an area’s involvement, we used a small volume correction (SVC) of 16 mm, *p*<0.01 corrected at voxel level, using co-ordinates given in a previous study [2].

## Results

### Behavioral data

Table 1 shows behavioral data collected in the scanning experiment. The proportion of stimuli which participants responded to as beautiful, indifferent or ugly in both visual and musical conditions is presented. Since we were interested in the experience subjects had over the entire 16 s of exposure to the stimuli, we specifically instructed them to respond only after the stimulus period ended. Subjects could respond anytime within the 5 s response period.

**Table 1:** Behavioral data collected in fMRI study.

Stimulus modality	Beautiful	Indifferent	Ugly
Visual	40.00%	25.09%	34.91%
(range)	(56.7–30.0)	(46.7–16.7)	(50.0–26.7)
Musical	42.24%	25.44%	32.32%
(range)	(58.7–30.0)	(43.3–13.3)	(43.7–23.3)

Distribution of behavioral ratings during the scanning experiment by stimulus modality, averaged over all subjects. Range shows maximum and minimum percentages among subjects.

### Beautiful > Ugly

**Figure 1:**
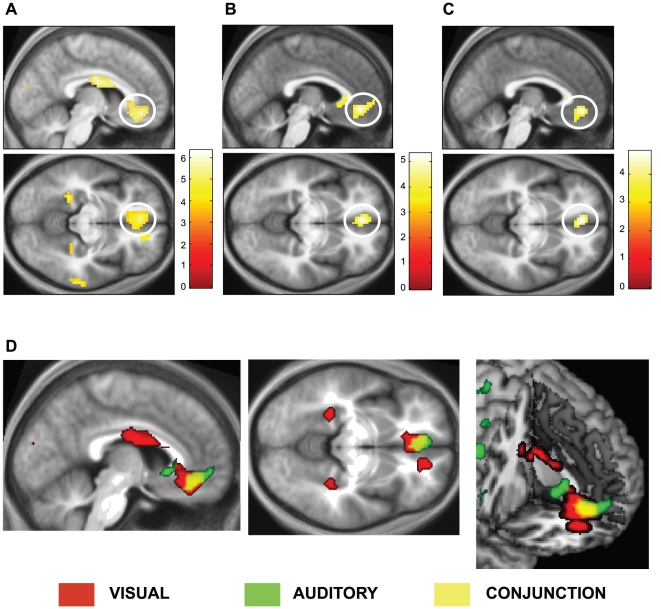
Cortical activation correlating with the experience of beauty. Statistical parametric maps rendered onto averaged anatomical sections (average of 21 subjects) showing the T statistic for the contrasts (A) *Visually Beautiful > Visually Ugly,* (B) *Musically Beautiful > Musically Ugly* and (C) the results of a conjunction analysis for *Visually Beautiful > Visually Ugly and Musically Beautiful > and Musically Ugly*. Upper row shows activity in mid-saggital sections and the middle row in horizontal sections of the brain. (D) shows the overlap in zones within the medial orbito-frontal cortex (mOFC) activated by visually beautiful (red), musically beautiful (green) stimuli, and the overlap between the two activations (yellow). Random effects analysis with 21 subjects. Display threshold *p*<0.001 (uncorrected). MNI co-ordinates of activation: A: at (−6 41–11). B: at (−3 41–8). And C: at (−3 41–8). The co-ordinates in D are the same as in C.

Our chief interest was to determine the cortical activity that correlates with experiences that were qualified as beautiful or ugly by the subjects. We therefore used the following contrasts: *(a)* Beautiful > Ugly for visual and musical stimuli; *(b)* Beautiful > Not Beautiful, that is, Beautiful > Indifferent + Ugly for visual and musical stimuli; *(c)* Beautiful > Indifferent for visual and musical stimuli. The results of these contrasts are given in Table 2, which shows that (*a*) the contrasts Visually Beautiful > Visually Ugly led to activation in the mOFC, at −6 41–11, while Musically Beautiful vs. Musically Ugly led to activation in mOFC at −3 41–8 (Figure 1); (*b*) the contrasts Visually Beautiful > Visually Indifferent + Visually Ugly led to activation in the mOFC, at 3 35–11 and −3 38–11 (with the application of a 16 mm SVC), while the contrast Musically Beautiful > Musically Indifferent + Musically Ugly led to activation in the OFC at 0 38–5 (with the application of a 16 mm SVC); *(c)* the contrasts Visually Beautiful > Visually Indifferent led to activation in the mOFC at 6 32–5 (with the application of a 16 mm SVC), while the contrast Musically Beautiful > Musically Indifferent led to activation in the mOFC at 3 38–5 (with the application of a 16 mm SVC).

**Table 2:** Activated areas correlating with the experience of beauty.

Brain regions	L/R	x	y	z	T	kE
**Visually Beautiful > Visually Ugly**						
Caudate nucleus	L	−9	−1	25	6.33	208
Medial OFC	L	−6	41	−11	5.42	178
**Musically Beautiful > Musically Ugly**						
Medial OFC	L	−3	41	−8	5.32	83
**Visually Beautiful > Indifferent + Ugly**						
Medial OFC (SVC)	R	3	35	−11	5.13	102
Medial OFC (SVC)	L	−3	38	−11	4.89	102
Caudate nucleus	L	−12	−1	28	5.27	92
Caudate nucleus	L	−6	20	−5	5.11	92
**Musically Beautiful > Indifferent + Ugly**						
Medial OFC		0	38	−5	5.12	11
**Visually Beautiful > Indifferent**						
Medial OFC (SVC)	R	6	32	−5	3.70	26
**Visually Ugly > Indifferent**						
*No suprathreshold clusters*						
**Musically Beautiful > Indifferent**						
Medial OFC (SVC)	R	3	38	−5	3.17	21
**Musically Ugly > Indifferent**						
Supra marginal gyrus	R	66	−34	34	6.72	101

Location, MNI co-ordinates, cluster size and values for the activations produced by the contrasts: *Visually Beautiful > Visually Ugly, Musically Beautiful > Musically Ugly, Visually Beautiful > Visually Indifferent + Ugly, Musically Beautiful > Musically Indifferent + Ugly, Visually Beautiful > Visually Indifferent, Visually Ugly > Visually Indifferent, Musically Beautiful > Musically Indifferent and Musically Ugly > Musically Indifferent.* In this and subsequent tables, all activations are cluster level significant at p<0.05 (corrected), although some of these were also significant at voxel level. Where we had a priori knowledge of an area's involvement, we applied a small volume correction (SVC) of 16 mm indicated as SVC.

The mOFC was the only cortical area that was commonly activated by all these contrasts, although each contrast also showed other activations (summarized in Table 2). This naturally led us to the heart of our enquiry, which was to learn, through the application of a conjunction analysis, whether activity in the same part of the mOFC correlated with the experience of visual and musical beauty. We used the following contrast for our conjunction analysis: [Visually Beautiful > Visually Ugly] and [Musically Beautiful > Musically Ugly]. This led to a significant conjunction in the mOFC at −3 41–8 (*p*<0.05, corrected) using an SVC of 16 mm. The results are given in Table 3. Superimposing the activations derived from the contrast Visually Beautiful > Visually Ugly and Musically Beautiful > Musically Ugly (using MRIcron: http://www.cabiatl.com/mricro), showed that the areas of activation derived from the two contrasts overlap substantially, if not totally.

**Table 3:** Activation area in the conjunction analysis.

Brain regions	L/R	x	y	z	T	kE
**Visually Beautiful + Musically Beautiful > Visually Ugly + Musically Ugly**						
Medial OFC	L	−6	41	−11	7.17	1153
Medial OFC	L	−3	26	4	5.38	1153
Caudate nucleus	L	−12	−1	25	5.30	126
**[Visually Beautiful > Visually Ugly] and [Musically Beautiful > Musically Ugly]**						
Medial OFC (SVC)	L	−3	41	−8	4.81	54

Table 3. Activations for the contrast *Visually Beautiful + Musically Beautiful > Visually Ugly + Musically Ugly* and conjunction analysis for *Visually and Musically Beautiful > Visually and Musically Ugly.*

Thus the only common area activated by stimuli that were judged to be beautiful, regardless of whether they were visual or musical, was located in the mOFC. We refer to this area as subdivision A1 of the mOFC (see Figure 1 and Discussion).

To check for the possibility that it may take longer to comprehend or experience beauty derived from one source compared to the other, we analyzed the data from one representative subject further with respect to different times within the viewing period. The data from this subject was divided into 4 periods, corresponding to 4, 8, 12, and 16 s after stimulus onset. Analyzing data from each time segment separately using boxcar functions, we found activity in mOFC with musical stimuli in the first 3 segments while with visual stimuli the activity in mOFC was detected with the last three segments (*p*<0.001 uncorrected). Hence the mOFC was active during most of the period of stimulus presentation for both visual and musical stimuli even if the activation induced by musical beauty was slightly earlier (4 s) than that induced by visual beauty. We conclude that the 16 s model that we have used with boxcar function is an appropriate model in this study.

### Strength of activation in mOFC

**Figure 2:**
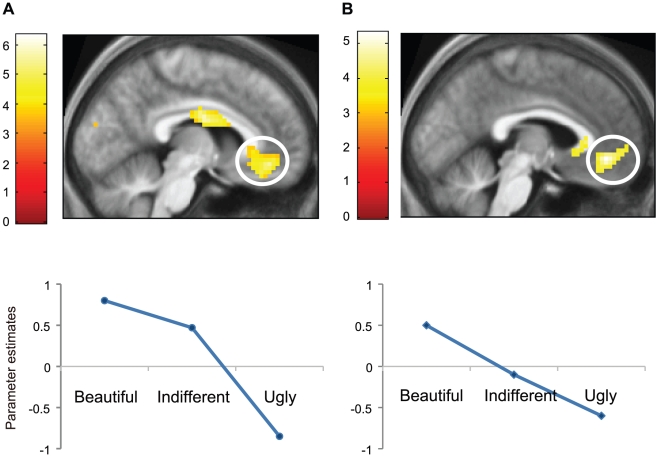
Modulation of cortical activity by aesthetic rating. Averaged parameter estimates showing modulation by beauty rating (Beautiful, Indifferent and Ugly) in mOFC for (A) visual stimuli (at −6 41–11) and (B) musical stimuli at −3 41–8. A linear relationship with beauty rating was observed in both conditions.

We wanted to learn whether the strength of activation in the mOFC is proportional to the strength of the declared experience of beauty, when viewing or listening to visual and musical stimuli. A parametric analysis of the relationship between intensity of experience and the BOLD signal showed that the activity was parametrically modulated within the mOFC, for visual stimuli at −6 41–11 (*p* =  0.03) and for musical stimuli, with the application of a 16 mm SVC, at −3 41–11 (*p* = 0.003) (Figure 2).

### Other activations in the contrast Beautiful > Ugly

Besides the mOFC, the body of the caudate nucleus, which has been shown to be active in a variety of emotional states, including the viewing of a loved romantic partner [10] and the experience of beauty [3], was also significantly active (Table 2).

### Ugly > Beautiful

The inverse contrast, Ugly > Beautiful, led to activation in a number of areas, summarized in Table 4. The contrast Visually Ugly > Visually Beautiful led to activation in left and right amygdala at −18–4 −14 and 36 2–11; in visual cortex at 42-40-20, corresponding to the right fusiform gyrus; at -24-97-11, corresponding to the left inferior occipital gyrus and in the left superior medial frontal gyrus at -9 62 25. With the application of a 16 mm SVC, left somato-motor cortex at -42 -10 61 and left postcentral gyrus at -51-16 52 showed significant activation. The former was close to the activation site for ugly stimuli in the study by Kawabata and Zeki (2004) [2].

**Table 4:** Activated areas correlating with the experience of ugliness.

Brain regions	L/R	x	Y	z	T	kE
**Visually Ugly > Visually Beautiful**						
Amygdala	L	−18	−4	−14	7.87	471
Amygdala	R	36	2	−11	6.45	446
Fusiform gyrus	R	42	−40	−20	6.22	385
Inferior occipital gyrus	L	−24	−97	−11	5.71	549
Superior medial gyrus	L	−9	62	25	5.76	118
Somato-motor cortex (SVC)	L	−42	−10	61	5.00	16
Postcentral gyrus (SVC)	L	−51	−16	52	4.48	16
**Musically Ugly > Musically Beautiful**						
*No suprathreshold clusters*						

Activations for the contrasts *Visually Ugly > Visually Beautiful, Musically Ugly > Musically Beautiful.*

There was no activity at the corrected significance level in the contrast of Musically Ugly > Musically Beautiful. The application of a conjunction analysis using the contrast Visually Ugly > Visually Beautiful vs. Musically Ugly > Musically Beautiful did not give any significant activation.

### Quantitative relationship between experience of ugliness and cortical activation

**Figure 3:**
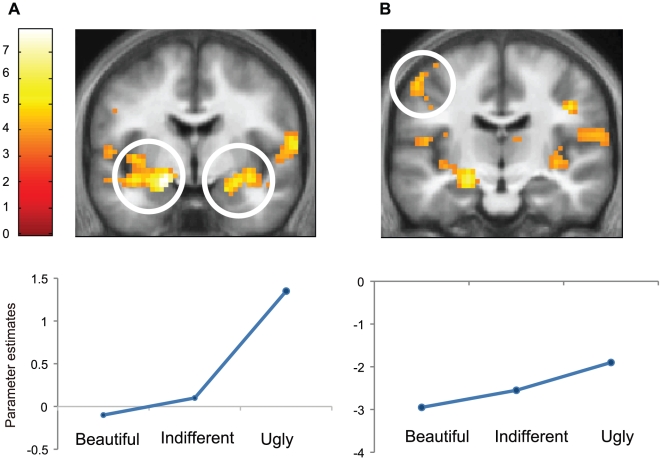
Cortical activations correlating with the experience of ugliness. Statistical parametric maps rendered onto averaged coronal anatomical sections (the average of 21 subjects) showing the T statistic for the contrasts Visually *Ugly > Visually Beautiful*. Random effects analysis with 21 subjects: display threshold *p*<0.001(uncorrected). (A) Coronal planes showing activations in left and right amygdala (−18 −4 −14) and (36 2 −11). (B) Left somato-motor cortex (−42 −10 61 SVC). Averaged parameter estimates showing modulation by beauty rating (Beautiful, Indifferent and Ugly) in left amygdala and left somato-motor cortex. A linear relationship with beauty rating was observed at each voxel.

Parametric analysis for visual stimuli showed a negative linear relationship between BOLD signal and declared intensity of experiences at the most significant voxels in left and right amygdala at −24 −4 −17 and 27 −4 −14 and in visual cortex at −24 −97 −11, corresponding to left inferior occipital cortex. With the application of a 16 mm SVC, a similar relationship was also observed in left postcentral gyrus −51 −16 52 (*p*<0.01, corrected) (Figure 3).

A corresponding analysis for musical stimuli did not show any significant activation.

## Discussion

### Activity in mOFC during the experience of beauty

The mOFC is a large expanse of cortex that has several architectonic areas (including Brodmann areas 10, 11, 12, 32 and 25). It apparently receives few direct sensory inputs but has strong connections with the basal ganglia [11] and its subdivisions are also heavily interconnected [12]. The mOFC has been activated in many studies probing the relationship of reward, pleasure and judgment [7], as well as the experience of beauty [2]–[4], [6] and value [13], to cortical activity. Given the variety of conditions that have been reported to lead to activation of mOFC, our principal interest was to determine whether the same or different subdivisions of this large expanse of cortex are active during the experience of beauty derived from different sources. The approach that we have adopted is similar to the one that we used to determine the cortical activity that correlates with the experience of temporally asynchronous patterns derived from different sources [14]. That result showed that each of the sources for temporal asynchrony activates a different zone of frontal cortex, with an additional, common, zone activated by all. In this study, the experience of beauty derived from visual and musical sources correlated with much the same part of the mOFC, the overlap between the two being extensive and possibly total.

### Relationship to previous studies

Many, if not all, studies that have addressed the neural correlates of the experience of beauty have found activity in mOFC, although sometimes the region is referred to otherwise. For example, Vartanian and Goel 2003 [3] refer to their site of activation as being in the anterior cingulate or the sub-genual anterior cingulate although their locus of activation, at −10 42 −6, is close to the locus in the study of Kawabata and Zeki (2004) [2], that of Kirk et al. (2009) [15] and this one. Similarly, Tsukiura and Cabeza (2011) attribute their locus of activation in response to facial attractiveness and moral goodness to anterior cingulate but the site of activation, at −4 44 1 [6], is very close to the one reported in this study and, in our view, belongs more appropriately to mOFC. The activation site reported by Di Dio et al (2009) in the supplementary material to their study of beauty is in the mOFC at (−6 36 −6 and 8 52 −6) [4] as is the activity reported by Kranz and Ishai (2006) [16], Cloutier et al. (2008) [17] and O'Doherty et al. (2003) [18] for facial attractiveness. The activations in all these studies fall well within field A1 of mOFC as outlined in the Results section. As well, studies of the relationship of value to cortical activity have also implicated the mOFC [13]. Even the study of Jacobsen et al. (2006), which differed somewhat from the studies mentioned above in that it involved judgments of beauty vs. symmetry, reported activation in the mOFC, though at a somewhat more dorsal level (at 1 23 32 and 1 54 26) [19]. There is one exception to this list which is the result derived from use of magnetoencephalography (MEG) (e.g. [20]). This may possibly have been due to the fact that activity in medially situated cortex is not easily detectable by MEG.

In sum, a great many results are in agreement that the experience of beauty correlates with activity in mOFC. To avoid any ambiguity and to relate the area demarcated here to areas of mOFC implicated in other studies, especially those related to judgment, evaluation, reward and desire, we tentatively refer to the area we have described as field A1 of mOFC. It is because of this apparent agreement, that field A1 of mOFC is active in most studies that have explored the relationship between cortical activity and the experience of beauty, that we concentrate on it in this discussion. The extent and boundaries of A1 must at present be tentative. We place its center at −3 41 −8 and estimate it to have a diameter of between 15–17 mm. There may be further functional subdivisions within it.

Taking our current results, as well as all the above studies, into account, we conclude (*a*) that the experience of beauty derived from visual and musical sources correlates with activity in the mOFC; (*b*) that, within the mOFC it correlates more specifically with activity in field A1; and (*c*) that the experience of beauty derived from at least two modalities, visual and musical, shares a common cortical locus in field A1 of mOFC. We therefore modify Burke's 1757 definition given above and say that ‘*Beauty is, for the greater part, some quality in bodies that correlates with activity in the mOFC by the intervention of the senses*’.

### Field A1 of mOFC, value and judgment

The paradigm that we used in this study is, inevitably, both judgmental and evaluative and it therefore makes it interesting to discuss our results in relation to axiology and to previous results that have explored the relationship of value to brain activity. We agree with DW Gotshalk [21] that “beauty is a value”, that it commonly evokes desire and that whatever is desired has value, although we tend to place beauty more in the perceiver than in the object, without denying that objects may have characteristics that qualify them as beautiful to one or many subjects. This essentially implies that there must be an intimate link in the cortical processing that is linked to value, desire and beauty. It is therefore interesting to note that the activity in A1 of mOFC that we report here is almost co-terminous with the activity reported in previous studies of the neural correlates of desire [22] and of value judgments [13]. This in turn not only reflects what is well known about the relationship of value, judgment, beauty and desire in axiology and philosophical discourse generally but also implies that there might be a value assigning system in the brain that is either supra-modal, that is to say not linked to value within any particular domain, or has specializations within it related to different values (see below).

It is interesting to note in this context that the judgments that we speak of above relate to positive judgments, strongly linked to reward and pleasure. We did not find activity in A1 of mOFC that correlates positively with the experience of ugly stimuli, although ugliness, too, involves a judgment. Instead, the parametrically modulated activity with the experience of ugliness was confined to the amygdala and left somato-motor cortex. This implies that there may be a functional specialization within the brain for at least two different kinds of judgment, those related to positive, rewarding, experiences and those related to negative ones. Future studies may yet reveal further specializations for judgments in different domains.

### Other activations

#### A: Visual and auditory cortex

The contrasts Visually Beautiful > Musically Beautiful led to widespread activity within visual cortex, while the contrast Musically Beautiful > Visually Beautiful led to widespread activity within auditory cortex. That such a large expanse of visual or auditory cortex should have been active is not surprising because the stimuli, whether visual or auditory, had many different characteristics; for example, the visual stimuli consisted of portraits, landscapes, still lifes and were in color while the musical stimuli had different degrees of melody, and harmony, and some were derived from large-scale orchestral performances while others from smaller ones. The activation of these sensory areas in conjunction with activation of mOFC is important for the theory we advance below.

#### B: Caudate nucleus

One of the more interesting activations was in the caudate nucleus, which was also activated in previous studies charting the neural correlates of emotional states [23], [24]. The caudate activations reported here have two features: (a) their location is similar to the location of the activity observed in previous studies of beauty [3] and in studies of the neural correlates of romantic love [10], [24], [25], and (b) the activation in it is proportional to the intensity of the declared experience of beauty. This close juxtaposition constitutes an interesting neural commentary on the traditional emphasis made in world literature on the relationship between love and beauty. Another interesting point about caudate activity is that it is evident only during the experience of visual beauty, with no parallel activation during the experience of musical beauty. We have no current explanation for this.

### Linear relationship between strength of cortical activity and strength of declared experience of beauty

Confirming previous studies from this and other laboratories [2], [3], [18], the activity in the mOFC was parametrically modulated, the BOLD signal being higher for stimuli rated as beautiful than those rated neutral or ugly. This was also true for the caudate nucleus, though only during the experience of visual beauty. A conjunction analysis using results derived from both auditory and visual scans once again showed that the same region (A1) of mOFC was parametrically modulated by both visual and musical stimuli, thus adding further to the conclusion that activity in one and the same brain area correlates in the same way with the experience of beauty derived from these two different sources. The experience of visual stimuli as ugly, on the other hand, correlated with activity in the amygdala and (with the application of an SVC) in left somato-motor cortex, among other areas (see Table 4). This activity, too, was proportional to the declared intensity of the experience. When we searched for quadratic modulation, we could not find increased activity in amygdala during the experience of both beauty and ugliness. Indeed, we could not detect any areas that had a quadratic relationship with the stimuli (i.e. were active during the experience of beautiful and ugly, but not indifferent, stimuli). In this, our results differ from those of Winston et al. (2007) who found that attractive and unattractive faces, but not ones judged to be neutral, lead to amygdala activation [26]. The reason for this difference is not known.

Taken together, these results imply that the subjective experience of beauty and of ugliness can be objectively ascertained and measured.

### Toward a brain-based definition of beauty

Taking the two principal results of this study, namely that activity in a single region (field A1) of mOFC correlates with experience of both visual and musical beauty and that there is a linear relationship in it between the BOLD signal and the declared intensity of the experience of beauty, leads us towards the formulation of a brain based definition of beauty.

The question of what beauty is has resisted adequate definition for centuries. Some, such as Vitruvius, Alberti and Leonardo Da Vinci, have sought to understand beauty in terms of the characteristics of the apprehended object. In visual art and architecture this may be reduced to symmetry, proportion, harmony and so on, while in music it may be beat, harmony and rhythm. But what are the characteristics that confer beauty on a more complex scene, such as a theatrical, operatic or cinematic one? And what would the characteristics of moral beauty be?

An issue that has much exercised philosophers of art and aesthetics and intrudes into any discussion of beauty is the relationship of beauty to art. While art has been traditionally associated with beauty in the popular mind as well as in past philosophical and artistic speculation, the notion that art and beauty can be equated has of course been questioned in the past and received a fatal blow when Marcel Duchamp presented his urinal, which he euphemistically named *The Fountain*, to an art exhibition; it then received a further blow with his *Readymades*, which Duchamp considered to constitute “art without an artist”. Notions of art have since changed and many will today acknowledge that something considered to be a work of art need not be perceived as beautiful, good examples being some of the paintings of Francis Bacon, or the nudes of Lucian Freud, which is not to say that these works do not have considerable artistic merit both in their painterly style and in projecting truths, including truths about decay and ugliness. But any work, be it considered art or not, may be subjectively experienced as being beautiful by an individual. This leads us to divorce art from beauty in this discussion and concentrate on beauty alone. In our study, we were essentially indifferent to whether a stimulus, be it visual or auditory, constituted a work of art, our only concern being with whether the individual subject, in the scanner, experienced the work as being beautiful or not.

In trying to provide an answer, we have been inspired by a critical question asked by the English art historian, Clive Bell, in his book entitled *Art* [27], though less so by the answer he gave. Bell was concerned in the main with visual beauty but we extend our argument to beauty in general. He wrote, “If we can discover some quality common and peculiar to all the objects that provoke it [beauty], we shall have solved what I take to be the central problem of aesthetics”. Unlike Hume, who placed beauty entirely in the perceiver, Bell searched for that “peculiar quality” in the apprehended objects while also giving primacy to the perceiver. He wrote: “All systems of aesthetics must be based on personal experience–that is to say, they must be subjective” [27]. What quality, he asked, “is common to Sta Sophia and the windows at Chartres, Mexican sculpture, a Persian bowl, Chinese carpets, Giotto's frescoes at Padua and the masterpieces of Poussin, Piero della Francesca, and Cézanne?”, a list that excludes music. We modify his question slightly by adding music and asking: what was common to all the beauty experiences that each of our subjects had when viewing the different visual and musical stimuli? Our results inspire us to provide, speculatively and tentatively, and perhaps even provocatively, a new, and neurobiological, answer to Bell's question as modified by us, an answer based exclusively on the perceiver rather than on the object, which is not to say that objects may not have characteristics that qualify them as beautiful.

The answer Bell gave is that the single characteristic that defines all works of art is “significant form”. Such a definition has many drawbacks, chief of which is defining what significant form might be in painting, music, fashion, design, film, opera and the many other areas in which we experience beauty, including moral beauty. Indeed, Bell himself was vague about what “significant form” might be in terms of even elementary visual attributes such as color and line. The term, being resistant to a definition that applies to all areas in which we experience beauty, thus also becomes impossible to measure and quantify. We therefore propose instead a neurobiological definition that makes it un-necessary to define “significant form” or indeed any other characteristic of the work being apprehended, a definition that is amenable to measurement and quantification and which relies on the perceiver alone. We propose that all works that appear beautiful to a subject have a single brain-based characteristic, which is that they have as a correlate of experiencing them a change in strength of activity within the mOFC and, more specifically, within field A1 in it. Our proposal shifts the definition of beauty very much in favor of the perceiving subject and away from the characteristics of the apprehended object and gives added strength to the Latin proverb that “*De gustibus non est disputandum*” (in matters of taste there is no dispute). We emphasize again that we do not wish to imply that objects that are classified as beautiful do not have certain characteristics that aid in this classification, although what these characteristics are has been, and continues to be, a subject of debate.

Our definition thus not only distinguishes sharply between artistic merit and aesthetic value but is also indifferent to what is art and what is not art. Almost anything can be considered to be art, but only creations whose experience has, as a correlate, activity in mOFC would fall into the classification of beautiful art. That the activity in the mOFC is proportional to the intensity of beauty experienced gives added strength to our theory, since the strength of activation is related to the intensity of the experience alone, regardless of the extent to which the work can be classified as a work of art or not. A painting by Francis Bacon may be executed in a painterly style and have great artistic merit but may not qualify as beautiful to a subject, because the experience of viewing it does not correlate with activity in his or her mOFC. The definition we propose takes aesthetics very much into the subjective, though quantifiable, arena: it applies only to an individual at a specific time and place since what is judged and experienced as beautiful at one moment and in one context by one subject may not be so experienced by another in a different context. Put differently, for an individual who experiences beauty in a Francis Bacon painting, with a concomitant change in activity within mOFC, the work can be qualified as beautiful to that individual. Our definition thus makes it un-necessary to consider other factors such as up-bringing, culture, context, connoisseurship and monetary value in the definition of what constitutes the aesthetic appeal of a work of art, although all these factors may contribute to the experience of beauty. Indeed, it is for this very reason that we included people from different cultures and ethnic backgrounds in our pool of subjects. There are of course many iconic works of art, such as the music of Beethoven or the *Pietà* of Michelangelo, which are experienced as beautiful by those who belong to different cultures, backgrounds and ethnic groups. This may be accounted for, as Immanuel Kant did in his *Critique of Judgment* [28], by supposing the existence of a *sensus communis,* that is to say a brain organization that is similar across individuals and cultures, which such works stimulate. We are currently addressing this in greater detail.

We are of course aware that activity in mOFC correlates with the experience of pleasure and reward, whether real or imagined, and its expectation [29]–[31]. This naturally raises, at a neurobiological level, an issue long discussed in the humanities, namely the relationship of aesthetic experience to pleasure (see Graham Gordon, *The Philosophy of Art* [32]). It can be argued that Wagner's Prelude to *Tristan und Isolde* is infinitely more subtle and beautiful than a composition by, for example, a rock artist. But this argument has more to do with what is art and what is not art than with what is perceived as beautiful and rewarding and what is not. Many who admire and are rewarded by listening to rock music, which they find beautiful, will probably have little time for Wagner, and vice versa. We would expect that, in subjects who find rock music rewarding and beautiful, their experience of the beauty of rock music will correlate with activity in their mOFC. Our definition is concerned with what an individual subject experiences as beautiful at a given moment, nothing else.

It is interesting to note that, contrary to the experience of beauty, we could not locate, through our conjunction analyses, a common area in which activity correlated with the experience of musical and visual ugliness, a negative finding for which we have no current explanation.

### Co-activation of mOFC and perceptive areas

One objection to our hypothesis is that, currently, activity in mOFC may be related to other experiences, such as judgment, evaluation, decision-making and reward in other domains, ones that are not directly related exclusively to beauty. For the sake of clarity and because of the complex architectonic configuration of mOFC, we designate the area that was active in this study as division A1 of mOFC. Activation of mOFC in other reward-related tasks, such as monetary reward, involves a different overall pattern of brain activation than the one we report here. Moreover, such reward tasks may or may not activate field A1 of mOFC. A recent study [33] reported overlapping activation with juice and monetary rewards in a region corresponding to A1 of mOFC, although the results of that study, being based on either uncorrected statistics at *p*<0.005 or corrected statistics at *p*<0.05 but with the use of an 8 mm SVC, are somewhat weak and require further study. This is especially so, since another study based on money rewards puts activity in the orbito-frontal cortex outside A1 [34] and at a significantly more anterior position than in the study of Kim et al. Hence the need for a more precise definition of the relationship of activations derived from different kinds of reward tasks to the extent of field A1 of mOFC.

In fact a specialization within mOFC may be conferred on it by the cortical route taken to it. In our study, although only activity in one cortical area, A1 of mOFC, correlated with the experience of musical and visual beauty, the path to mOFC through the two domains was different. With musical experience of beauty, auditory areas of the brain were co-active with A1 of mOFC while we could not detect any activity in the caudate nucleus. With experience of visual beauty, the caudate nucleus was very much co-active with A1 of mOFC as were the visual areas (we use the term co-active because the temporal limitations of the fMRI method do not allow us to isolate the sequence of activity in these areas). Hence, basing ourselves more on Burke's definition of beauty given above, as one mediated by the senses, we consider that it is not activation of mOFC alone that is a determinant of beauty; it is rather the co-activation of field A1 of mOFC with the specialized sensory and perceptive area, or areas, and possibly (in the case of visual stimuli) with the caudate nucleus as well. Hence we broaden our neurobiological definition of beauty given above to include not only activation of mOFC but also its co-activation with sensory areas that feed it. The interaction between these sensory areas, and other regions such as the caudate, and A1 of mOFC, and how activity in the latter is modulated by activity in the former remains a very interesting puzzle for the future.

We emphasize that our theory is tentative; there are many other experiences that may be deemed to be beautiful besides the visual and musical. Our theory will stand or fall depending upon whether future studies of the experience of beauty in other domains show that, in these too, the experience correlates with activity in field A1 of mOFC.

## Supporting Information

Table S1 **Behavioral data collected in preliminary behavioral test.** Distribution of behavioral ratings during preliminary test by stimulus modality, averaged over all subjects. Range shows maximum and minimum percentages among subjects.
(DOCX)
