# Supplementary material for: Toward A Brain-Based Theory of Beauty
Source: PLoS One. 2011 Jul 6;6(7):e21852. doi: 10.1371/journal.pone.0021852 (PMC3130765; doi:10.1371/journal.pone.0021852)
Supplement: Table S1 — Behavioral data collected in preliminary behavioral test. Distribution of behavioral ratings during preliminary test by stimulus modality, averaged over all subjects. Range shows maximum and minimum percentages among subjects. (DOCX) [file pone.0021852.s001.docx]

Table S1. Behavioral data collected in preliminary behavioral test.

| Stimulus modality | 9-7 (beautiful) | 6-4 (indifferent) | 3-1 (ugly) |
| --- | --- | --- | --- |
| Visual | 41.28% | 26.07% | 32.65% |
| (range) | (55.3-32.2) | (44.7-19.6) | (49.9-25.3) |
| Musical | 40.17% | 28.49% | 31.34% |
| (range) | (56.1-30.3) | (46.6-15.1) | (43.0-23.7) |

Distribution of behavioral ratings during preliminary test by stimulus modality, averaged over all subjects. Range shows maximum and minimum percentages among subjects.
